# *Hansenula polymorpha* Aat2p is targeted to peroxisomes via a novel Pex20p‐dependent pathway

**DOI:** 10.1002/1873-3468.13168

**Published:** 2018-06-30

**Authors:** Ann S. Thomas, Arjen M. Krikken, Rinse de Boer, Chris Williams

**Affiliations:** ^1^ Molecular Cell Biology Groningen Biomolecular Sciences and Biotechnology Institute University of Groningen The Netherlands

**Keywords:** Aat2p, aspartate aminotransferase, *Hansenula polymorpha*, Peroxisome protein import, Pex20p

## Abstract

*Saccharomyces cerevisiae* Aat2p contains a peroxisomal targeting signal type‐1 and localizes to peroxisomes in oleate‐grown cells, but not in glucose‐grown cells. Here, we have investigated Aat2p from the yeast *Hansenula polymorpha*, which lacks a recognizable peroxisomal targeting signal. Aat2p tagged with GFP at its C terminus displays a dual cytosol‐peroxisome localization in ethanol‐grown cells. The partial peroxisomal localization of Aat2p persisted in the absence of the classical cycling receptors Pex5p and Pex7p but Aat2p targeting to peroxisomes was reduced in cells deleted for the matrix protein import factors PEX1, PEX2 and PEX13. Furthermore, we demonstrate that Aat2p targeting to peroxisomes requires Pex20p. Together, our data identify a Pex20p‐dependent pathway for targeting Aat2p to peroxisomes.

## Abbreviations

***Hp*Aat2p**, *Hansenula polymorpha* Aat2p

**PCR**, polymerase chain reactions

**PNS**, Post Nuclear Supernatant

**PTS**, peroxisome targeting signal

**Pyc‐1**, pyruvate carboxylase‐1

**TCA**, trichloroacetic acid

The peroxisome is a specialized, single membrane‐bound organelle that is ubiquitous in eukaryotic cells. Peroxisomes function in compartmentalizing a wide range of metabolic pathways, displaying a high degree of plasticity in their functions, depending on species and cell type 1. Some specific functions include glycolysis in trypanosomes and the production of penicillin in certain fungi while well‐conserved roles include the beta‐oxidation of fatty acids and hydrogen peroxide degradation 2. In humans, defects in genes involved in peroxisome biogenesis result in fatal disorders 3.

A robust yet adaptable import system is indispensable to maintain a steady flow of proteins to the peroxisomal matrix, because such proteins are produced in the cytosol and imported post‐translationally 4. Matrix proteins are targeted to peroxisomes via a Peroxisome Targeting Signal (PTS). The first identified PTS (PTS1) was in firefly luciferase 5. Further studies established that the PTS1 consists of a C‐terminal dodecamer sequence with a C‐terminal tripeptide harbouring the consensus sequence (SAC)‐(KRH)‐(LM) 6, 7. The PTS1 sequence is recognized by the receptor protein Pex5p 8, 9. Pex5p recruits cargo proteins to the peroxisomal matrix by means of a C‐terminal tetratricopeptide repeat domain, which interacts with the PTS1 sequence 10. The N‐terminal region of Pex5p is required for membrane docking and recycling. A second PTS (PTS2) was identified in a subset of peroxisomal matrix proteins, bearing the N‐terminal consensus sequence (R/K)(L/V/I)X_5_(H/Q)(L/A) 11, 12. The cognate receptor for this motif is Pex7p. However, unlike the PTS1 pathway, PTS2 import requires the Pex20p family of coreceptor proteins in yeast and fungi 13, 14 or a long isoform of Pex5p in higher eukaryotes 15. Pex7p functions in cargo binding, whereas the coreceptors are required for membrane docking and recycling. Evidence exists for a third pathway that is independent of a PTS1, but requires the N‐terminal domain of Pex5p 16, 17. Sometimes referred to as the PTS3 pathway, to date no PTS3 consensus sequence has been described 18. Proteins bearing no endogenous PTS such as Pnc1p and Cu/Zn superoxide dismutase may gain entry into peroxisomes by ‘piggybacking’ with other PTS containing peroxisomal matrix proteins 19, 20. Such examples demonstrate that the absence of a PTS does not exclude peroxisomal localization.

*Saccharomyces cerevisiae* has two aspartate aminotransferase isoenzymes called Aat1p and Aat2p. Aat1p is mitochondrial 21 while Aat2p contains a PTS1 and is localized to peroxisomes in oleic acid‐grown cells, while it is mainly cytosolic in glucose‐grown cells 22. Here, we demonstrate that *Hansenula polymorpha* Aat2p (*Hp*Aat2p), which lacks a recognizable PTS, displays dual localization to the cytosol and peroxisomes in glucose and ethanol‐grown cells, whereas it is cytosolic in methanol‐grown cells. In addition, we show that Aat2p targeting involves the matrix protein import factors Pex1p, Pex2p and Pex13p but is independent of the PTS receptor proteins Pex5p or Pex7p. Instead, Aat2p targeting to peroxisomes solely relies on the PTS2 coreceptor Pex20p. Thus, we identify a novel targeting pathway for matrix proteins that requires Pex20p.

## Materials and methods

The *H. polymorpha* strains, plasmids and oligonucleotides used in this study are described in Tables S1–S3 respectively.

### Construction of plasmids and strains

To construct pHIPH4, the hygromycin B resistance cassette was removed from pAG32 23 using NcoI (partial digestion) and EcoRV and cloned into pHIPZ4 24 digested with Asp718I (klenow‐treated) and NcoI, resulting in the plasmid pHIPH4.

The *AAT2* deletion cassette was constructed as follows: A PCR fragment of 1854 bp was amplified from plasmid pHIPH4 using primers ANN PR15 and ANN PR16. This cassette was transformed into *H. polymorpha yku80* cells and integration of the deletion cassette into the genome was confirmed using southern blotting.

The plasmid‐bearing *AAT2* downstream of the *AOX* promoter was constructed as follows: a 1.2 kb fragment corresponding to the *AAT2* gene was amplified from genomic DNA using primers ANN PR35 and ANN PR51. The PCR product was digested with HindIII and XbaI and ligated with HindIII XbaI‐digested pHIPN4 plasmid 25 resulting in the vector pHIPNP_*AOX*_ *‐AAT2* (pANN016).

To obtain the plasmid‐bearing *AAT2* downstream of its endogenous promoter, a 0.6 kb fragment upstream of the *AAT2* gene corresponding to the promoter region was amplified from genomic DNA using primers ANN PR83 and ANN PR84. The corresponding PCR product was digested with NotI and HindIII and ligated with NotI‐HindIII digested pANN016 plasmid, resulting in the vector pHIPNP_*AAT2*_‐*AAT2* (pANN015). The C‐terminal GFP fusion of Aat2p produced under control of the endogenous promoter was created by amplifying a 0.9 kb fragment of the *AAT2* gene from genomic DNA without the stop codon, followed by incorporation of the restriction sites HindIII and BglII using primers ANN PR27 and ANN PR28. The resulting PCR product was digested with HindIII and BglII and ligated into HindIII‐BglII‐digested pHIPZ‐Pex13mGFP 26 to obtain pHIPZ‐Aat2mGFP (pANN009). This plasmid was linearized with Acc651 (KpnI) prior to transformation into *H. polymorpha yku80, pex1.atg1, pex2, pex3, pex5.pex7, pex13, pex19* and *pex20* cells.

The plasmid for disruption of *ATG1* was constructed using Multisite Gateway technology (Thermo Fisher Scientific Corporation, Waltham, MA, USA) as follows. First, the 5′ and 3′ flanking regions of the *ATG1* gene were amplified by PCR using the primer pairs ARM PR 18, ARM PR 19 and ARM PR 16, ARM PR 17, respectively, using *H. polymorpha* NCYC495 genomic DNA as a template. The resulting fragments were then recombined into donor vectors pDONR P4‐P1R and pDONR P2R‐P3, resulting in plasmids pENTR *ATG1* 5′ and pENTR *ATG1* 3′ respectively. Both entry plasmids were recombined with the destination vector pDEST R4‐R3 together with entry plasmid pENTR221‐hph, resulting in plasmid pARM011.

To obtain a *pex1.atg1* double deletion strain, the *ATG1* deletion cassette corresponding to 2.6 kb was amplified from plasmid pARM011 using primers ARM PR20 and ARM PR21. This cassette was transformed into *H. polymorpha pex1* cells 27 and integration of the deletion cassette into the genome was confirmed with PCR using primers ARM PR62 and ARM PR63.

The plasmid pHIPN‐Pex14‐mCherry was linearized with XhoI prior to transformation into *H. polymorpha yku80, pex1.atg1, pex5.pex7* or *pex20* cells harbouring pANN009. Plasmid pHIPH‐Pex14‐mCherry was made by amplifying Pex14‐mCherry from plasmid pHIPN‐Pex14‐mCherry with PCR using primer PRARM001 and PRARM002. The resulting PCR product was digested with HindIII and NotI and ligated into HindIII‐NotI‐digested pHIPH4. The resulting plasmid pHIPH‐Pex14mCherry was linearized with BlpI prior to transformation into *H. polymorpha pex2* and *pex13* cells containing Aat2mGFP.

For the production of antibodies against Aat2p, the *AAT2* gene was amplified from genomic DNA along with NcoI and HindIII restriction sites using primers AAT2 Ab_F and AAT2 Ab_R. NcoI‐HindIII‐digested PCR fragment was used for ligation with NcoI‐HindIII‐digested pETM30 harbouring the GST‐His_6_ tag.

Transformation of *H. polymorpha* was performed by electroporation as described previously 28. Preparative polymerase chain reactions (PCR) for cloning were carried out with Phusion High‐Fidelity DNA Polymerase (Thermo Fisher Scientific Corporation). Initial selection of positive transformants by colony PCR was carried out using Phire polymerase (Thermo Fisher Scientific Corporation). DNA restriction enzymes were used as recommended by the suppliers (Thermo Fisher Scientific Corporation, New England Biolabs, Ipswich, MA, USA).

All integrations were confirmed by colony PCR and deletions were confirmed by southern blotting.

### Cultivation conditions

*Hansenula polymorpha* cells were grown in batch cultures at 37 °C on mineral media 29 supplemented with 0.25% glucose, 0.5% methanol or 0.3% ethanol as carbon source and 0.25% ammonium sulphate as nitrogen source. Leucine, when required, was added to a final concentration of 30 μg·mL^−1^. For growth on plates, YPD (1% yeast extract, 1% peptone and 1% glucose) media was supplemented with 2% agar. Resistant transformants were selected using 100 μg·mL^−1^ zeocin (Invitrogen, Grand Island, NY, USA), 100 μg·mL^−1^ nourseothricin (Werner Bioagents, Jena, Germany) or 200 μg·mL^−1^ hygromycin (Invitrogen).

### Biochemical techniques

Extracts prepared from cells treated with 12.5% trichloroacetic acid (TCA) were prepared for SDS/PAGE as detailed previously 30. Equal amounts of protein were loaded per lane. Blots were probed with mouse monoclonal antisera against GFP (sc‐9996, Santa Cruz Biotech, Heidelberg, Germany) or His tag (34660, Qiagen, Hilden, Germany) and rabbit polyclonal antisera against Aat2p (Fig. S1). Pyruvate carboxylase‐1 (Pyc‐1) 31 was used as the loading control.

### Sequence alignment of Aat2p

Multiple sequence alignments of protein sequences were generated using ClustalW2 (http://www.ebi.ac.uk/Tools/msa/clustalw2/) and visualized with GeneDoc (http://www.nrbsc.org/old/gfx/genedoc/). The following accession numbers were used: *Eremothecium gossypii* (NP_985758.1); *Kluyveromyces lactis* (XP_455876.1); *Candida glabrata* (XP_445496.1); *Candida albicans* (XP_711144.1); *Saccharomyces cerevisiae* (CAA97550.1); *Debaryomyces hansenii* (XP_459482.1); *Pichia kudriavzevii* (KGK39897.1); *Pichia membranifaciens* (XP_019016745); *Pichia pastoris* (XP_018213151); *Hansenula polymorpha* (XP_018213151); *Candida arabinofermentas* (ODV84999.1). *Coccidioides immitis* (XP_001240699); *Aspergillus nidulans* (XP_663652.1); *Aspergillus oryzae* (XP_001826273.1); *Aspergillus fumigatus* (XP_755298.1); *Neurospora crassa* (XP_962457.1); *Magnaporthe grisea* (XP_003719674.1); *Penicillium rubens* Wisconsin (XP_002565847); *Botrytis cinerea* (EMR86905); *Sclerotinia sclerotiorum* (XP_001585000.1).

### Fluorescence microscopy

All images were taken at room temperature using a 100x 1.30 NA Plan Neofluar objective. Wide‐field images were taken using a Zeiss Axioscope A1 fluorescence microscope (Carl Zeiss, Oberkochen, Germany). Images were taken using a Coolsnap HQ2 digital camera and micro manager software. A 470/40 nm bandpass excitation filter, a 495 nm dichromatic mirror and a 525/50 nm bandpass emission filter were used to visualize the GFP signal. DsRed fluorescence was visualized with a 546/12 nm bandpass excitation filter, a 560 nm dichromatic mirror and a 575/640 nm bandpass emission filter. A 587/25 nm bandpass excitation filter, a 605 nm dichromatic mirror and a 647/70 nm bandpass emission filter were used to visualize mCherry fluorescence.

### Electron microscopy

Cells were fixed in a mixture of 0.2% glutaraldehyde and 3% formaldehyde in 0.1 m cacodylate buffer pH 7.2 for 4 h on ice. Cells are embedded in unicryl (Aurion, 14660) and polymerized for 4 days under UV at 10 °C. Immunogold labelling was performed on 70 nm ultrathin sections using specific polyclonal antisera and gold conjugated goat anti‐rabbit antiserum (Aurion, 806.011). Sections were stained with a mixture of 0.5% uranyl acetate and 0.2% methylcellulose before viewing them in a Philips CM12 electron microscope.

### Cell fractionation

Cell fractionation experiments were performed as described in 32. Briefly, protoplasts were prepared using Zymolyase (Brunschwig Chemie, Amsterdam, the Netherlands) and subjected to homogenization using a Potter homogenizer. Cell debris was removed by centrifuging the homogenate twice at 3000 ***g*** for 10 min at 4 °C to obtain the Post Nuclear Supernatant (PNS). The PNS was centrifuged at 30 000 ***g*** for 30 min at 4 °C to separate the membrane pellet (P) from the soluble fraction (S).

## Results and Discussion

### Aat2p in *Hansenula polymorpha* lacks a recognizable PTS sequence

**Figure 1:**
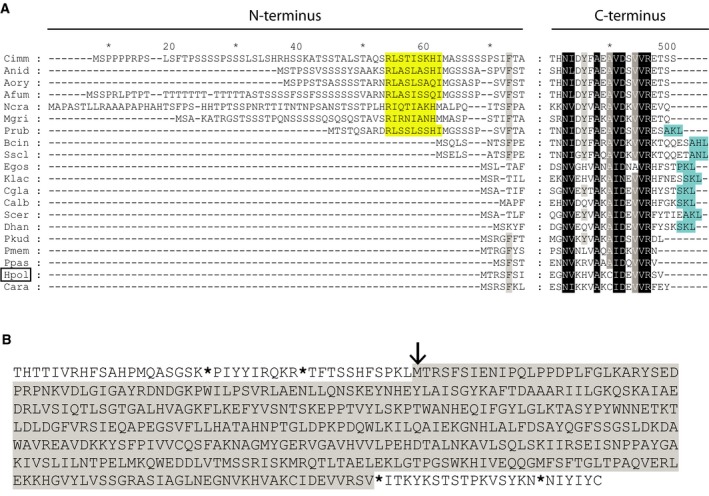
*Hp*Aat2p lacks a recognizable Peroxisomal Targeting Sequence. (A) Multiple sequence alignment of the N‐ and C‐terminal regions of Aat2p from different yeast and fungi. Black shading indicates identity. Similarity is indicated as white letters that are shaded dark grey when present in 19 of the 22 sequences and as black letters shaded light grey when present in 14 of the 22 sequences. Putative PTS1 sequences are shaded cyan, putative PTS2 sequences in yellow. (Fungi: Cimm, *Coccidioides immitis*; Anid, *Aspergillus nidulans*; Aory, *Aspergillus oryzae*; Afum, *Aspergillus fumigatus*; Ncra, *Neurospora crassa*; Mgri, *Magnaporthe grisea*; Prub, *Penicillium rubens* Wisconsin; Bcin, *Botrytis cinerea*; Sscl, *Sclerotinia sclerotiorum*. Yeasts: Egos, *Eremothecium gossypii*; Klac, *Kluyveromyces lactis*; Cgla, *Candida glabrata*; Calb, *Candida albicans*; Scer, *Saccharomyces cerevisiae*; Dhan, *Debaryomyces hansenii*; Pkud, *Pichia kudriavzevii*; Pmem, *Pichia membranifaciens*; Ppas, *Pichia pastoris*; Hpol (boxed), *Hansenula polymorpha*; Cara, *Candida arabinofermentas)*. (B) *Hansenula polymorpha* Aat2 protein sequence (shaded in grey) showing potential upstream and downstream amino acid residues. The start codon of Aat2p is indicated with an arrow, while stop codons are indicated with an asterisk.

Sequence alignment of yeast and fungal Aat2 proteins revealed that most contain either a PTS1 or PTS2 33, whereas Aat2p from *Penicillium rubens* displays both. However, *H. polymorpha* Aat2p does not possess a recognizable PTS, similar to Aat2p from several other yeast species (Fig. 1A). Recent reports demonstrate that translational read‐through, where the translation machinery skips a stop codon, can result in proteins containing a PTS1 34, 35. To analyse whether this is the case for Aat2p, we scanned the region downstream of the *AAT2* gene until the next stop codon. No PTS1 targeting sequence was encoded by the region preceding this stop codon (Fig. 1B). Likewise, the region upstream of Aat2p did not encode a PTS2 (Fig. 1B). These observations demonstrate that Aat2p from *H. polymorpha* does not possess canonical peroxisome targeting information in its protein sequence.

### *Hp*Aat2p partially localizes to peroxisomes

**Figure 2:**
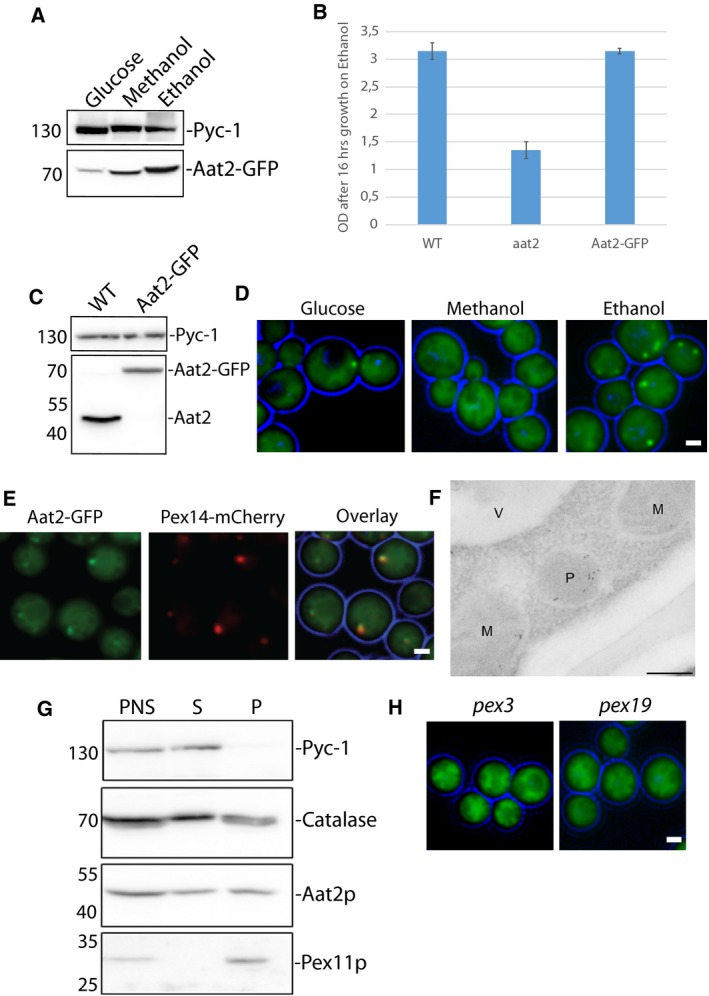
*Hp*Aat2p partially localizes to peroxisomes. (A) Cell lysates of WT cells grown on different carbon sources expressing Aat2p tagged with GFP at the C terminus were subjected to SDS/PAGE and immunoblotting using antibodies directed against GFP or Pyc‐1 (loading control). (B) Graph representing growth of WT,* aat2* or a strain harbouring Aat2‐GFP on ethanol containing media. Growth of cells is indicated as a measure of optical density of the culture at an absorbance of 660 nm. (C) Western blot showing WT or a strain containing Aat2‐GFP, probed with antibodies directed against Aat2p. (D) Fluorescence microscopy analysis of WT cells expressing Aat2p tagged with GFP at the C terminus, grown on various carbon sources. Scale bar represents 1 μm. (E) Colocalization analysis of Aat2‐GFP with the peroxisomal marker Pex14‐mCherry. Scale bar represents 1 μm. (F) Immuno‐Electron microscopy analysis showing WT cells grown on ethanol. Aat2p was labelled with antibodies against Aat2p and detected with goat anti‐rabbit antibodies conjugated to 6‐nm gold particles. M‐Mitochondria, P‐Peroxisomes, V‐Vacuole. Scale bar represents 200 nm. (G) Cell fractionation analysis of WT cells, displaying the postnuclear supernatant (PNS), supernatant (S) and organelle pellet (P) fractions probed with SDS/PAGE, western blotting and antibodies against the cytosolic protein Pyc‐1, the peroxisomal matrix protein Catalase, the peroxisomal membrane protein Pex11p and Aat2p. (H) Fluorescence microscopy analysis of ethanol‐grown *pex3* or *pex19* cells expressing Aat2‐GFP. Scale bars represent 1 μm. Note that all GFP fluorescent images were processed differently, in order to visualize the GFP signal optimally.

Aat2p from *S. cerevisiae* is cytosolic in cells grown on glucose but peroxisomal in oleate‐grown cells 22. We therefore investigated the behaviour of Aat2p in *H. polymorpha* cells grown on carbon sources which repress (glucose) or induce (ethanol, methanol) peroxisome proliferation 36. First, we produced a construct expressing Aat2p fused to GFP at the C terminus (Aat2‐GFP), under control of the *AAT2* promoter. These cells produced Aat2‐GFP as the only version of Aat2p. Western blot analysis revealed that in glucose‐grown cells the levels of the GFP fusion protein are lower compared to those in ethanol or methanol‐grown cells (Fig. 2A).

The deletion of *aat2* results in a growth defect on ethanol (Fig. 2B), indicating that Aat2p contributes to ethanol metabolism in *H. polymorpha*. Cells that express Aat2‐GFP grow comparably to the WT strain on ethanol (Fig. 2B), indicating that the Aat2‐GFP fusion protein is fully functional, although western blotting analysis using antibodies raised against Aat2p demonstrated that levels of Aat2‐GFP are lower than that of endogenous Aat2p in the WT strain (Fig. 2C), which could suggest that the GFP tag has an effect on Aat2p expression levels.

Next, we assessed the localization of Aat2‐GFP in cells grown on glucose, ethanol and methanol using fluorescence microscopy (Fig. 2D). Aat2‐GFP appeared mostly cytosolic in glucose‐grown cells, although spots were observed occasionally (Fig. 2D). Ethanol‐grown cells displayed pronounced accumulation of Aat2‐GFP in spots, although fluorescence was also detectable in the cytosol (Fig. 2D). The GFP signal appeared cytosolic in cells grown on methanol. Since ethanol‐grown cells showed the clearest accumulation of GFP‐tagged Aat2p in spots, we used this condition for further investigations into the localization of Aat2p. To determine whether GFP spots indeed corresponded to peroxisomes, the peroxisomal marker Pex14‐mCherry was introduced into cells producing Aat2‐GFP. GFP spots colocalized with Pex14‐mCherry (Fig. 2E), indicating that these GFP spots were indeed peroxisomes.

We also created a version of Aat2p tagged with GFP at the N terminus (GFP‐Aat2), under control of the *AAT2* promoter and introduced this construct into both WT cells and cells deleted for *AAT2*. In both cases, we observed GFP‐positive spots that colocalized with the peroxisomal marker DsRed‐SKL in cells grown on ethanol containing media (Fig. S2A) indicating that GFP‐Aat2 can target to peroxisomes, similar to Aat2‐GFP. However, we noticed that protein levels of GFP‐Aat2 were much lower than those of Aat2‐GFP (Fig. S2B), suggesting that the N‐terminal GFP tag strongly influences Aat2 protein expression. Therefore, we conducted further fluorescence microscopy analysis using Aat2‐GFP (see below).

To further investigate the peroxisomal localization of Aat2p, we performed immunolabelling experiments of WT cells using antibodies raised against Aat2p, observing that untagged Aat2p can localize to peroxisomes (Fig. 2F). We also occasionally observed the presence of gold particles in mitochondria (Fig. 2F), which may be cross‐reactivity of the Aat2p antibodies towards the mitochondrial aspartate aminotransferase Aat1p. Next, we investigated Aat2p localization using organelle fractionation experiments (Fig. 2G), demonstrating that Aat2p can be partially found in the pellet fraction, similar to the peroxisomal matrix protein Catalase and the peroxisomal membrane protein Pex11p.

Finally, we investigated the localization of Aat2‐GFP in *pex3* and *pex19* cells, which are devoid of normal peroxisomes. As can be expected for a peroxisomal matrix protein, GFP fluorescence invariably displayed a cytosolic pattern in these deletion strains (Fig. 2H). Taken together, our data indicate that Aat2p can target to peroxisomes in cells grown on ethanol, despite the fact that the protein lacks a bona‐fide PTS1 or PTS2 signal. The protein may display a dual localization, because GFP fluorescence was also detected in the cytosol of cells producing the Aat2‐GFP. However, we cannot exclude that the cytosolic localization is caused by the GFP tag.

### Pex1p, Pex2p and Pex13p play a role in targeting Aat2p to peroxisomes

**Figure 3:**
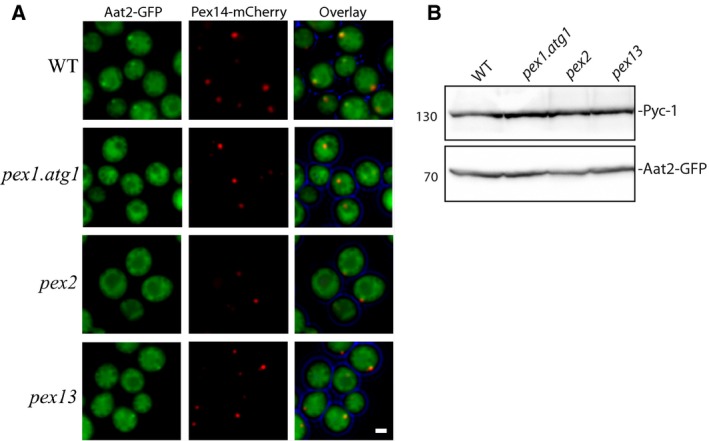
Pex1p, Pex2p and Pex13p play a role in targeting *Hp*Aat2p to peroxisomes (A) Fluorescence microscopy images of WT,* pex1.atg1, pex2* and *pex13* cells grown for 16 h on ethanol containing media. Besides the peroxisomal marker Pex14‐mCherry, all cells produced the fusion protein Aat2‐GFP. Scale bar represents 1 μm. (B) SDS/PAGE and immunoblot analysis of lysates from the deletion strains presented in (A) probed with antibodies directed against GFP or Pyc‐1 (loading control).

To determine if peroxisomal localization of Aat2p relies on components of the matrix import pathway, we investigated the localization of Aat2‐GFP in strains lacking *PEX1*,* PEX2* or *PEX13* (Fig. 3A). Pex13p is involved in docking of the cycling receptor proteins on the peroxisomal membrane 37 whereas the E3 ligase Pex2p plays a role in ubiquitinating the cycling receptors 38, which in turn acts as a signal for their removal from the peroxisomal membrane by the AAA‐ATPase Pex1p 39.

In strains lacking *PEX1* or *PEX2*, the number of GFP spots was dramatically reduced (Fig. 3A), suggesting a role for these proteins in targeting Aat2‐GFP to peroxisomes. We analysed the effect of deleting *PEX1* in an *atg1* background because *PEX1* deletion results in the autophagic degradation of peroxisomal ghosts 40. In *pex13* cells, we observed that although less pronounced than in WT cells, Aat2‐GFP spots could still be seen in certain cells (Fig. 3A), which could indicate that Pex13p is involved in targeting Aat2p to peroxisomes, but is not essential. Western blot analysis revealed that Aat2‐GFP was produced in all strains (Fig. 3B). Taken together, our results suggest that Pex1p, Pex2p and, to a lesser extent Pex13p, are involved in targeting Aat2p to peroxisomes.

### *Hp*Aat2p targeting to peroxisomes depends on Pex20p

**Figure 4:**
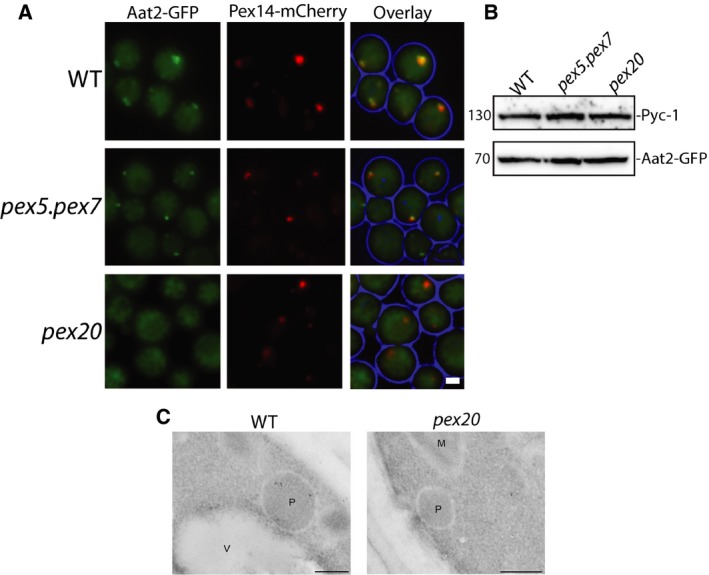
*Hp*Aat2p targets to peroxisomes in a Pex20p‐dependent manner. (A) Fluorescence microscopy images of WT,* pex5.pex7* and *pex20* deletion strains grown for 16 h on ethanol containing media. Besides the peroxisomal marker Pex14‐mCherry, all cells produced the fusion protein Aat2‐GFP. Scale bar represents 1 μm. (B) SDS/PAGE and immunoblot analysis of lysates from the deletion strains presented in (A) probed with antibodies directed against GFP or Pyc‐1 (loading control). (C) Immuno‐Electron microscopy analysis showing WT and *pex20* cells grown on ethanol for 16 h. Aat2p was labelled with anti‐Aat2p and detected with goat anti‐rabbit antibodies conjugated to 6‐nm gold particles. M‐Mitochondria, P‐Peroxisomes, V‐Vacuole. Scale bar represents 200 nm.

Finally, we investigated how Aat2p is transported to peroxisomes. To achieve this, we followed Aat2‐GFP in a strain lacking Pex5p and Pex7p, the cycling receptors for the PTS1 and PTS2 pathways respectively. FM analysis demonstrated that Aat2‐GFP colocalizes with the red peroxisomal membrane marker Pex14‐mCherry in the absence of both receptor proteins (Fig. 4A), indicating that the targeting of Aat2‐GFP to peroxisomes is independent of Pex5p and Pex7p.

We next investigated the role of Pex20p in Aat2p targeting. Pex20p binds to Pex7p and acts as a coreceptor for PTS2 proteins. It functions in the docking and recycling steps during PTS2 import, whereas Pex7p is predominantly involved in binding to the PTS2 signal. Interestingly, we found that Aat2‐GFP became fully cytosolic in *pex20* deletion cells (Fig. 4A). Western blot analysis demonstrated that the Aat2‐GFP fusion protein was produced in all strains (Fig. 4B). Immuno‐EM analysis of WT and *pex20* cells grown on ethanol demonstrated that Aat2p was no longer peroxisomal in *pex20* cells (Fig. 4C). Together, these data indicate that Pex20p is required for targeting Aat2p to peroxisomes.

## Concluding remarks

Here, we have investigated Aat2p from the yeast *H. polymorpha*, demonstrating that Aat2p can localize to peroxisomes despite lacking a recognizable PTS. Our fluorescence microscopy data strongly suggest that Pex20p is involved in the targeting of Aat2p to peroxisomes in a Pex7p‐independent manner, indicating that Pex20p, like Pex5p and the recently described Pex9p 41, 42, could be a peroxisomal import receptor protein in its own right. Interestingly, a direct role for Pex20p in targeting proteins to the peroxisome has been suggested before, based on the observation that *H. polymorpha* Pex20p can interact directly with the PTS2 sequence 43. Furthermore, Pex20p from the yeast *Yarrowia lipolytica* binds to the PTS2 protein thiolase in a PTS2‐independent manner 44. This, coupled with the fact that Pex7p has not been identified in *Y. lipolytica* to date, would support our suggestion that Pex20p may directly act as a peroxisomal import receptor protein in certain organisms. To investigate further the role of Pex20p in targeting Aat2p to peroxisomes, we initiated *in vitro* binding experiments using purified Aat2p and Pex20p and checked for an interaction using pull‐down assays. We did not observe an interaction between the proteins. Perhaps Aat2p and Pex20p interact indirectly and require the presence of additional factors.

While it is clear that Aat2p can target to peroxisomes, the data presented here do not answer the question of whether Aat2p resides inside peroxisomes or whether it associates with the outside of the peroxisome. The fact that we identify roles for Pex1p, Pex2p and, to a lesser extent Pex13p, in Aat2p targeting to peroxisomes would suggest that Aat2p can gain access to the peroxisomal matrix, since these proteins are implicated in the import of many matrix proteins. However, further study is required to validate this.

The GFP‐tagged Aat2p shows partial peroxisomal localization in ethanol‐grown cells, while it is cytosolic in cells grown on methanol. Pex20p levels in cells grown on methanol are lower than those grown on glucose 43, which could suggest that Pex20p levels play a determining role in Aat2p localization. Similarly, levels of the import receptor Pex9p are regulated in a condition‐specific manner 41, 42, demonstrating that the localization of proteins to peroxisomes can be regulated by receptor protein abundance. Since cells adapt the function of peroxisomes to meet metabolic requirements, regulating peroxisomal targeting through receptor protein abundance would provide an additional level at which cells could determine peroxisome content and hence peroxisome function.

Finally, targeting of Aat2p to peroxisomes does not require Pex5p or Pex7p, the classical matrix protein import receptors, strongly suggesting that Aat2p possesses an as‐yet unidentified PTS that enables the protein to be recruited to peroxisomes. Future work aimed at characterizing the requirements for targeting Aat2p to peroxisomes will lead to a better understanding of how Aat2p achieves its peroxisomal localization and may also lead to the identification of additional proteins which have so far been excluded from the cohort of peroxisomal matrix proteins.

## Supporting information


**Table S1.** *H. polymorpha* strains used in this study.
**Table S2.** Plasmids used in this study.
**Table S3.** Oligonucleotides used in this study.
**Fig. S1.** Specificity of *H*pAat2p antibodies.
**Fig. S2.** N‐terminally tagged GFP‐Aat2 partially localizes to peroxisomes.
