# Supplementary material for: Hansenula polymorpha Aat2p is targeted to peroxisomes via a novel Pex20p‐dependent pathway
Source: FEBS Lett. 2018 Jun 30;592(14):2466–75. doi: 10.1002/1873-3468.13168 (PMC6099311; doi:10.1002/1873-3468.13168)
Supplement: Supplementary file 1 — Table S1. H. polymorpha strains used in this study. Table S2. Plasmids used in this study. Table S3. Oligonucleotides used in this study. Fig. S1. Specificity of HpAat2p antibodies. Fig. S2. N‐terminally tagged GFP‐Aat2 partially localizes to peroxisomes. [file FEB2-592-2466-s001.pdf]

Supporting information

Hansenula polymorpha Aat2p is targeted to peroxisomes via a novel Pex20p dependent pathway

Ann S. Thomas[a], Arjen M. Krikken[a], Rinse de Boer[a] and Chris Williams[a]

[a]Molecular Cell Biology, Groningen Biomolecular Sciences and Biotechnology Institute, University of Groningen, Nijenborgh 7, 9747AG Groningen, The Netherlands

[#]Corresponding author (c.p.williams@rug.nl)

Supporting materials and methods

Protein purification and preparation of antibodies

H. polymorpha Aat2p with a cleavable His_6-GST tag was produced in *E. coli* BL21 (DE3) RIL. Cells were grown at 37°C to an OD600 of 0.6 in Terrific Broth (TB) medium, transferred to 21°C and grown until an OD600 of 1.5. Gene expression was induced with 0.05mM IPTG (Invitrogen) for 16 h and cells were harvested by centrifugation. Cell pellets were resuspended in lysis buffer (50mM Tris-HCl pH 7.5, 150mM NaCl, 1% glycerol, 1mM DTT, 1mg/ml lysozyme, 10µg/ml DNase) and passed two times through a French press. Cell debris was removed by centrifugation and lysates were loaded onto glutathione sepharose-4B resin (GE Healthcare) pre-equilibrated with lysis buffer. The resin was extensively washed with lysis buffer and His_6-GST tagged proteins were eluted using lysis buffer containing 20mM reduced glutathione. The GST tag was cleaved from the protein using TEV protease and samples were passed over a Ni-NTA column. The concentrated protein sample was subjected to gel filtration using a Superdex 200 (16/60) column (GE Healthcare) equilibrated with 25mM Tris, 150mM NaCl, 1mM 2-mercaptoethanol, pH 7.5. The presence of purified

Aat2p was confirmed using SDS-PAGE and protein samples were sent for antibody production (Eurogentec). The properties of the resulting anti-Aat2p antibodies are shown in Figure S1.

Construction of strains containing N-terminally tagged GFP-Aat2

The N-terminal GFP fusion of Aat2p produced from its endogenous promoter was obtained by amplifying a 0.7 kb fragment of GFP along with the restriction sites HindIII and BamHI using pHIPZ-Pex13mGFP as template and the primers ANN PR46 and ANN PR47. Similarly, a fragment of 1.3 kb corresponding to *AAT2* was amplified incorporating BamHI and XbaI restriction sites from genomic DNA using primers ANN PR48 and ANN PR49. HindIII-BamHI digested GFP was ligated into HindIII-BamHI digested pUC19 and BamHI-XbaI digested *AAT2* was ligated with BamHI-XbaI digested pUC19. Following this, GFP was excised using HindIII and BamHI, *AAT2* was excised using BamHI and XbaI and these fragments were used in a three-way ligation with HindIII-XbaI digested pHIPN5 GFP-SKL [1] to yield pHIPN5-mGFPAat2 (pANN017). To produce the fusion protein from the endogenous promoter, the GFP-*AAT2* fragment was excised with HindIII and NsiI and ligated with HindIII-NsiI digested pANN015, resulting in the plasmid pHIPNP$_{AAT2}$-mGFPAat2 (pANN014). StuI was used for linearization before integration into *H. polymorpha yku80* and *aat2* cells.

The plasmid pHIPX7DsRed-SKL was linearized with DraI prior to transformation into WT+GFP-AAT2 and *aat2*+GFP-AAT2 cells.

Table S1: *H. polymorpha* strains used in this study.

Strains	Characteristics	Reference
yku80 (WT)	NCYC495, *leu1.1 YKU80::URA3*	[2]
WT+AAT2-GFP	Y*KU80::URA3* + pANN009	This study
pex1	NCYC495, *leu1.1 PEX1::URA3*	[3]
pex1.atg1	NCYC495, *leu1.1 PEX1::URA3, ATG1::HPH*	This study
pex2	NCYC495, *leu1.1 PEX2::URA3*	[4]
pex3	NCYC495, *leu1.1 PEX3::URA3*	[5]
pex5 (LEU2 marker, used for construction of pex5.pex7)	NCYC495, *ura3 PEX5::LEU2*	[6]
pex5.pex7	Segregant of cross between *PEX5::LEU2* and *PEX7::URA3*	This study
pex13	NCYC495, *leu1.1 PEX13::URA3*	[4]
pex19	NCYC495, *leu1.1 PEX19::URA3*	[7]
pex20	NCYC495, *leu1.1 PEX20::URA3*	[8]
WT+AAT2-GFP+Pex14-mCherry	Y*KU80::URA3* + pANN009+pHIPN-Pex14-mCherry	This study
pex1.atg1+AAT2-GFP+Pex14-mCherry	*PEX1::URA3,ATG1::HPH*+ pANN009+pHIPN-Pex14-mCherry	This study
pex2+AAT2-GFP+Pex14-mCherry	*PEX2::URA3*+ pANN009+pHIPH-Pex14-mCherry	This study
pex5.pex7+AAT2-GFP+Pex14-mCherry	*PEX5::LEU2,PEX7::URA3*+ pANN009+pHIPN-Pex14-mCherry	This study
pex13+AAT2-GFP+Pex14-mCherry	*PEX13::URA3*+ pANN009+pHIPH-Pex14-mCherry	This study
pex20+AAT2-GFP+Pex14-mCherry	*PEX20::URA3* + pANN009+pHIPN-Pex14-mCherry	This study
aat2	*AAT2::HPH*	This study
WT+GFP-AAT2	Y*KU80::URA3* + pANN014	This study
WT+GFP-AAT2+DsRed-SKL	Y*KU80::URA3* + pANN014+pHIPX7-DsRed-SKL	This study
aat2+GFP-AAT2	*AAT2::HPH*+ pANN014	This study
aat2+GFP-AAT2+DsRed-SKL	*AAT2::HPH*+pANN014+pHIPX7-DsRed-SKL	This study

Table S2: Plasmids used in this study.

Plasmid	Description	Reference
pAG32	*S. cerevisiae* vector containing hygromycin B resistance cassette (Hph^R), Amp^R	[9]
pHIPH4	pHIP vector bearing the *AOX* promoter, Amp^R, Hph^R	This study

pHIPZ4	pHIP vector containing the *AOX* promoter, Amp^R, Zeo^R	[10]
pHIPN4	Plasmid bearing the *AOX* promoter, Amp^R, Nat^R	[11]
pANN016	*AAT2* expressed from the *AOX* promoter, contains Amp^R, Nat^R	This study
pHIPZ-Pex13mGFP	*PEX13* expressed from the endogenous promoter with C-terminal GFP fusion, contains Amp^R, Zeo^R	[12]
pANN009	*AAT2* expressed from the endogenous promoter with C-terminal GFP fusion, contains Amp^R, Zeo^R	This study
pHIPN-Pex14-mCherry	*PEX14* expressed from the endogenous promoter with C-terminal mCherry fusion, contains Amp^R, Nat^R	[12]
pHIPH-Pex14-mCherry	PEX14 expressed from the endogenous promoter with C-terminal mCherry fusion, contains Amp^R, Hph^R	This study
pANN015	*AAT2* expressed from the endogenous promoter, contains Amp^R, Nat^R	This study
pDONR P4-P1R	Multisite Gateway vector; Kan^R, Cm^R	Invitrogen
pDONR P2R-P3	Multisite Gateway vector; Kan^R, Cm^R	Invitrogen
pENTR *ATG1* 5’	pDONR P4-P1R with 5’ flanking region of *ATG1*; Kan^R	This study
pENTR *ATG1* 3’	pDONR P2R-P3 with 3’ flanking region of *ATG1*; Kan^R	This study
pDEST-R4-R3	Multisite Gateway donor vector; Amp^R, Cm^R	Invitrogen
pENTR221-hph	pDONR 221 with *HPH*; Hph^R, Kan^R	[2]
pARM011	Plasmid bearing the *ATG1* deletion cassette, contains Amp^R ,Hph^R	This study
pETM30 AAT2	His_6GST-Aat2, for expression in *E. coli*; Kan^R	This study
pHIPN5GFP-SKL	GFP-SKL expressed from the *AMO* promoter, contains Amp^R, Nat^R	[1]
pANN017	*AAT2* expressed from the *AMO* promoter with N-terminal GFP fusion, contains Amp^R, Nat^R	This study
pANN014	*AAT2* expressed from endogenous promoter, with N-terminal GFP fusion, contains Amp^R, Nat^R	This study
pHIPX7DsRed-SKL	DsRed-SKL expressed from the constitutive *TEF* promoter, contains Kan^R, LEU2	[13]

Table S3: Oligonucleotides used in this study.

Primer	Sequence
ANN PR15	GATTTTGCCCTCTGTCAGGCTCGCCGAGAACCTGTTGCAGAACTC CAAGGCCCACACACCATAGCTTCAA
ANN PR16	TCGTCCTCCCACTGTTTCATCAGCTCGGGCGTGTTGAGGATCAGA GACACCGTTTTCGACACTGGATGGC

ANN PR27	CACGTAAAGCTTACAGACTCGTGTCTATCCAG
ANN PR28	GATACGAGATCTCACACTTCTCACCACCTCGTC
ANN PR35	GCGCAAGCTTATGACAAGATCCTTCAGCATCGAGAACATCC
ANN PR51	CGCTCTAGACTACACACTTCTCACCACCTCGTCAATACAT
ARM PR 16	GGGGACAGCTTTCTTGTACAAAGTGGCCGCCACAAATGGTGAAG TCGATC
ARM PR 17	GGGGACAACTTTGTATAATAAAGTTGCATCGAGCTTCTCGTTGCC CGTGAC
ARM PR 18	GGGGACAACTTTGTATAGAAAAGTTGGGCTGGAGAACGCGGCAG ATCC
ARM PR 19	GGGGACTGCTTTTTTGTACAAACTTGGGGAGGGGAAGGGTACCT CTC
ARM PR 20	ACAGGTCGTTGGTGACTTTAC
ARM PR 21	CTTCTCGTTGCCCGTGACC
ARM PR 62	AGGCCGTTCGGTTATGATAG
ARM PR 63	GGACAATGGCCGCATAACAG
PRARM001	ATAGCGGCCGCTTGCAGGAAGTCGACGAAAT
PRARM002	CGGAAGCTTTTACTTGTACAGCTCGTCCA
AAT2 Ab_F	CATGCCATGGCAATGACAAGATCCTTCAGCATCG
AAT2 Ab_R	CCCAAGCTTCTACACACTTCTCACCACCTCG
ANN PR83	AAGCGGCCGCGATTTTCGGGTCCAGAGTGT
ANN PR84	GCAAGCTTGGGGGAGAAATGGGAGGAAG
ANN PR 46	CGCAAGCTTATGGTGAGCAAGGGCGAG
ANN PR 47	GCGCGGATCCCTTGTACAGCTCGTCCATG
ANN PR48	GCGCGGATCCACAAGATCCTTCAGCATCGAG
ANN PR49	GCGCTCTAGACTACACACTTCTCACCACC

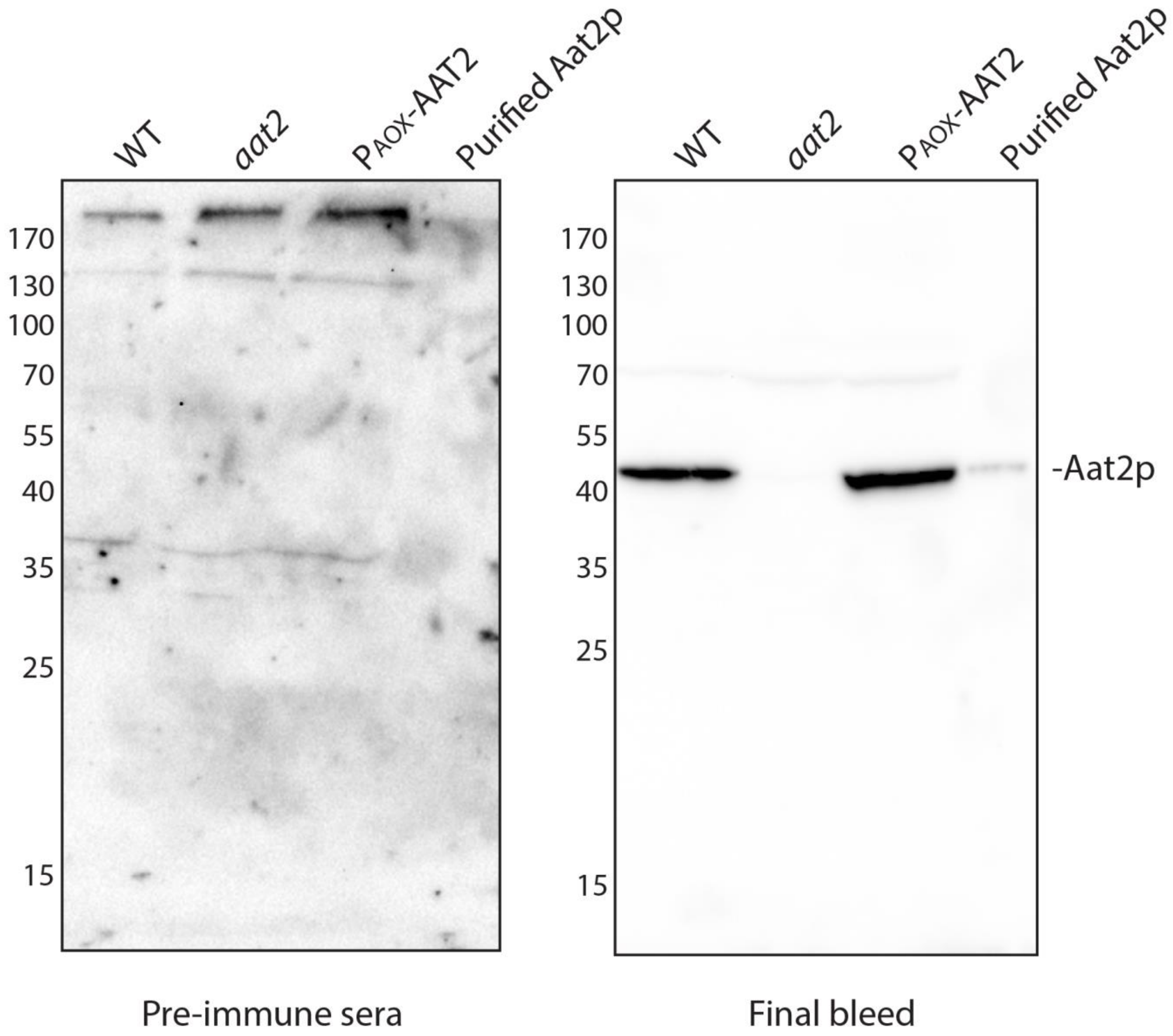

Figure S1: Specificity of *Hp*Aat2p antibodies. Western blot analysis of cell lysates from methanol grown WT cells, *aat2* cells and WT cells containing *AAT2* under control of the strong alcohol oxidase promoter (P_{AOX}-*AAT2*), together with purified Aat2p from *E.coli*, probed with pre-immune serum (left panel) or serum from a rabbit immunogenized with purified Aat2p (right panel). Blots were exposed for 20 sec.

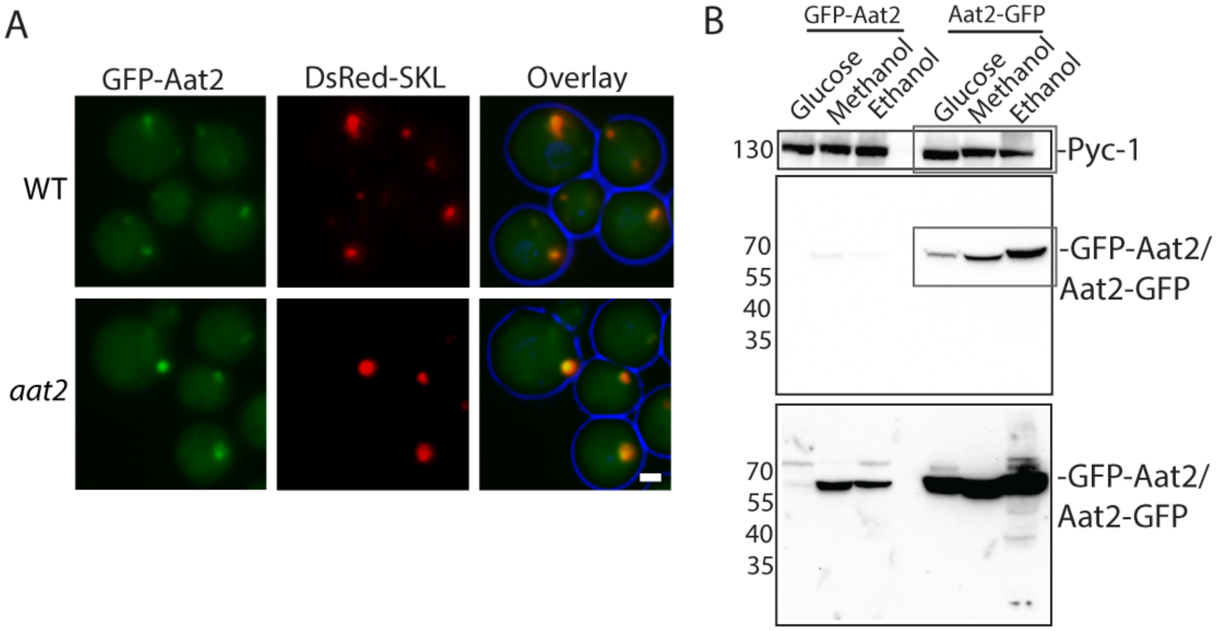

Figure S2: N-terminally tagged GFP-Aat2 partially localises to peroxisomes. (A) Co-localisation analysis of GFP-Aat2 in ethanol-grown WT (upper panel) or *aat2* (lower panel) cells co-expressing the peroxisomal matrix marker DsRed-SKL. Scale bar represents 1μm. **(B)** Cell lysates of WT cells grown on different carbon sources expressing Aat2p tagged with GFP either at the N- or C-terminus were subjected to SDS-PAGE and immunoblotting using antibodies directed against GFP or Pyc-1 (loading control). The two blots represent different exposures: top panel, 10 sec. exposure, bottom panel, 1000 sec. exposure. Part of this blot (boxed) is shown in Figure 2A.

Supporting references

1. Thomas, A. S., Krikken, A. M., van der Klei, I. J. & Williams, C. (2015) Phosphorylation of Pex11p does not regulate peroxisomal fission in the yeast Hansenula polymorpha, *Scientific reports.* **5**, 11.
2. Saraya, R., Krikken, A. M., Kiel, J. A., Baerends, R. J., Veenhuis, M. & van der Klei, I. J. (2012) Novel genetic tools for Hansenula polymorpha, *FEMS Yeast Res.* **12**, 271-8.
3. Kiel, J. A., Hilbrands, R. E., van der Klei, I. J., Rasmussen, S. W., Salomons, F. A., van der Heide, M., Faber, K. N., Cregg, J. M. & Veenhuis, M. (1999) Hansenula polymorpha Pex1p and Pex6p are peroxisome-associated AAA proteins that functionally and physically interact, *Yeast.* **15**, 1059-78.
4. Koek, A., Komori, M., Veenhuis, M. & van der Klei, I. J. (2007) A comparative study of peroxisomal structures in Hansenula polymorpha pex mutants, *FEMS Yeast Res.* **7**, 1126-33.
5. Baerends, R. J., Rasmussen, S. W., Hilbrands, R. E., van der Heide, M., Faber, K. N., Reuvekamp, P. T., Kiel, J. A., Cregg, J. M., van der Klei, I. J. & Veenhuis, M. (1996) The *Hansenula polymorpha PER9* gene encodes a peroxisomal membrane protein essential for peroxisome assembly and integrity, *J Biol Chem.* **271**, 8887-8894.
6. Van der Klei, I. J., Hilbrands, R. E., Swaving, G. J., Waterham, H. R., Vrieling, E. G., Titorenko, V. I., Cregg, J. M., Harder, W. & Veenhuis, M. (1995) The Hansenula polymorpha PER3 gene is essential for the import of PTS1 proteins into the peroxisomal matrix, *J Biol Chem.* **270**, 17229-17236.
7. Otzen, M., Perband, U., Wang, D., Baerends, R. J., Kunau, W. H., Veenhuis, M. & Van der Klei, I. J. (2004) Hansenula polymorpha Pex19p is essential for the formation of functional peroxisomal membranes, *J Biol Chem.* **279**, 19181-90.
8. Otzen, M., Wang, D., Lunenborg, M. G. & van der Klei, I. J. (2005) Hansenula polymorpha Pex20p is an oligomer that binds the peroxisomal targeting signal 2 (PTS2), *J Cell Sci.* **118**, 3409-18.
9. Schell-Steven, A., Stein, K., Amoros, M., Landgraf, C., Volkmer-Engert, R., Rottensteiner, H. & Erdmann, R. (2005) Identification of a novel, intraperoxisomal pex14-binding site in pex13: association of pex13 with the docking complex is essential for peroxisomal matrix protein import, *Mol Cell Biol.* **25**, 3007-18.
10. Salomons, F. A., Kiel, J. A., Faber, K. N., Veenhuis, M. & van der Klei, I. J. (2000) Overproduction of Pex5p stimulates import of alcohol oxidase and dihydroxyacetone synthase in a Hansenula polymorpha Pex14 null mutant, *J Biol Chem.* **275**, 12603-12611.
11. Cepinska, M. N., Veenhuis, M., van der Klei, I. J. & Nagotu, S. (2011) Peroxisome fission is associated with reorganization of specific membrane proteins, *Traffic.* **12**, 925-37.
12. Knoops, K., Manivannan, S., Cepinska, M. N., Krikken, A., Kram, A. M., Veenhuis, M. & Van der Klei, I. J. (2014) Preperoxisomal vesicles can form in the absence of Pex3p, *J Cell Biol* **204** 659–668.
13. Krikken, A. M., Veenhuis, M. & van der Klei, I. J. (2009) Hansenula polymorpha pex11 cells are affected in peroxisome retention, *FEBS J.* **276**, 1429-39.
